# Process concepts and analysis for co-removing methane and carbon dioxide from the atmosphere

**DOI:** 10.1038/s41598-023-44582-w

**Published:** 2023-10-12

**Authors:** Devesh Sathya Sri Sairam Sirigina, Aditya Goel, Shareq Mohd Nazir

**Affiliations:** 1 Department of Chemical Engineering, KTH Royal Institute of Technology, 11428 Stockholm, Sweden; 2 Department of Chemical Engineering, Columbia University, New York, NY 10027 USA; 3 Department of Chemical Engineering, Birla Institute of Technology and Science, Pilani — Goa Campus, Sancoale, Goa 403726 India

**Keywords:** Carbon capture and storage, Chemical engineering, Energy

## Abstract

Methane is the second largest contributor to global warming after CO_2_, and it is hard to abate due to its low concentration in the emission sources and in the atmosphere. However, removing methane from the atmosphere will accelerate achieving net-zero targets, since its global warming potential is 28 over a 100-year period. This work presents first-of-its-kind process concepts for co-removal of methane and CO_2_ that combines the catalytic conversion of methane step (thermal/photo-catalytic) with CO_2_ capture. Proposed processes have been analyzed for streams with lean methane concentrations, which are non-fossil emissions originating in the agricultural sector or natural emissions from wetlands. If the proposed processes can overcome challenges in catalyst/material design to convert methane at low concentrations, they have the potential to remove more than 40% of anthropogenic and natural methane emissions from the atmosphere at a lower energy penalty than the state-of-the-art technologies for direct air capture of CO_2_.

## Introduction

The Conference of Parties 26 (COP26) meeting recognized the necessity of mitigating non-CO_2_ greenhouse gases (GHGs), especially methane, to slow the warming in the next couple of decades^1^. Just in the last decade, the non-CO_2_ GHGs, in particular methane and nitrous oxide, were responsible for more than 0.5 °C rise in the temperature^2^. From a sectorial perspective, agriculture is the highest contributing sector to methane and nitrous oxide emissions in the world^3^. Among the non-CO_2_ GHG gases, methane is the highest contributor accounting for approximately two-thirds of the total non-CO_2_ GHG emissions. The anthropogenic methane emissions constitute about two-third of total methane emissions, with the wetlands dominating the natural source of emissions^4^. Natural sources of methane emissions, according to US EPA^4^, include wetlands (northern high-latitude and tropical); upland soils and riparian areas; oceans, estuaries, and rivers; permafrost; lakes; gas hydrates; geological sources; terrestrial arthropods (termites); and wild animals. Anthropogenic methane emissions include both fossil and non-fossil methane emissions. The sources of anthropogenic non-fossil methane emissions include enteric fermentation, landfills, manure management, wastewater treatment, flooded agricultural fields and incomplete combustion^5^. Incomplete combustion refers to the methane release from its usage for domestic purposes (cooking and heating in developing countries), burning of agricultural residues, and deforestation to use the land for agricultural purposes.^5^. Fossil methane, the chief constituent of natural gas, is released through leakages and venting during its extraction, transport and use in addition to emissions via ventilation air in coal mines^5^.

**Figure 1:**
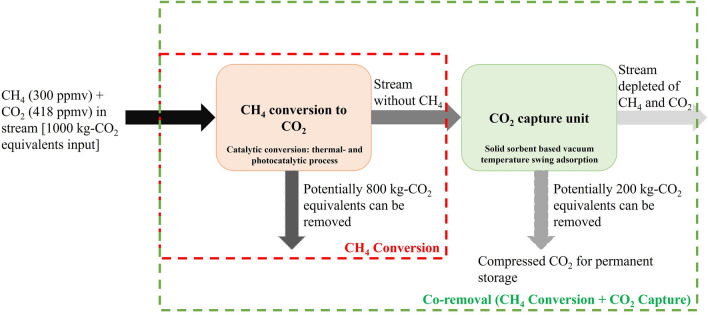
Representative schematic for the potential of removing CO_2_-equivalents from the atmosphere via methane conversion or co-removal of methane and CO_2_. An example for a low concentration methane stream, like air from stable ventilation in agricultural farms, is used in this case.

The agricultural sector is responsible for ⁓40% of anthropogenic methane emissions^6^. The energy sector is the next major sector contributing to about 40% of total anthropogenic CH_4_ emissions^7^. The rest of the emissions are from waste sector, and biomass burning^7^. A significant portion of the methane emissions from agriculture are attributed to enteric fermentation. Other sources within agriculture include manure management, rice cultivation, burning of crop residues, savanna fires, fires in humid tropic forests and organic soils, and on farm energy use^8,9^. The majority of methane emissions from energy sector are fossil-based with the emissions from operations related to oil, natural gas, and coal^7^. Emissions from bioenergy (incomplete combustion) account for a minor portion from the energy sector. Wetlands are responsible for ⁓40% of natural methane emissions^6^. It is estimated that around 70% of the emissions from energy sector can be mitigated through the implementation of existing technologies (methane utilization, flaring, leak detection and repair etc.)^7^. The measures to mitigate methane emissions from agriculture are also widely addressed in various literature^10–12^. However, in the case of agriculture most literature is focused on preventive measures, i.e., before the release of methane into the atmosphere. In the context of achieving net-zero greenhouse gas emission targets it is also important to deploy measures that remove residual methane emissions from the atmosphere. These emissions are associated with low concentrations and are hard to abate. The current work presents process routes to remove methane from low concentration streams by converting it to CO_2,_ and its possible integration with CO_2_ capture. Figure 1 summarizes the proposed concept for a stream having a total of 1000 kg CO_2_-equivalents coming in the form of 300 ppmv methane (methane concentration in the air from stable ventilation) and CO_2_ concentration in ambient air. By oxidizing/converting methane into CO_2_ results in removing 800 kg CO_2_-equivalents in this case, while a further 200 kg CO_2_-equivalents can be potentially removed by CO_2_ capture and permanent storage. There is an advantage of just converting methane to CO_2_ since the benefit of removing CO_2_ equivalents from the atmosphere can be achieved instantly. In addition, since the methane oxidation reaction is exothermic, the heat of the reaction can be utilized within the process, thereby reducing the overall energy penalty. This approach is different than concentrating or capturing methane from low concentration sources, where the energy penalty in process is recovered by utilizing the heating value of methane at a later stage. The multifaceted benefits of non-fossil methane mitigation from agriculture spanning beyond environment was shown in our previous work using sustainable development goals as the framework^13^.

Methane is a short-lived greenhouse gas with an atmospheric lifetime of 12 years in comparison to centuries for CO_2_^14^. On a 100-year timescale, the global warming potential (GWP) of methane is 28 times that of CO_2_^15^. On oxidation and without the capture of CO_2_, the cumulative radiative forcing from that parcel of methane over a 100-year period can be reduced by ~ 90%. In other words, just the conversion of 1 ton of methane to CO_2_ is 25 times more effective than removing 1 ton of CO_2_ from the atmosphere. Therefore, our focus is on proposing processes that can convert methane into CO_2_ from streams having < 1%-vol of methane. This conversion process is catalytically driven owing to low concentration of methane and its oxidation at lower temperatures than the auto-ignition temperature^16,17^. The development of methane catalytic conversion technologies has focused on the one hand on improving the reactor design, e.g., reversed flow and monolith reactors, and on the other, identifying the right catalyst^18,19^. Two types of catalysts have been researched upon: thermal and photo-catalysts. Pd supported on alumina has been the most reported thermal catalyst that can completely oxidize low concentration methane at > 300 °C^20^. Other possible catalysts systems reported in literature are based on Ni, Co, Mn^21,22^. N_2_O decomposition, in the presence of methane and excess oxygen by selective catalytic reduction (SCR) over Fe ion-exchanged zeolites, occurs at temperatures between 250 and 300 °C^23,24^. The catalyst systems were used in applications where temperature of the feed streams is already high, like in exhaust gases from vehicles, or where the required temperature is achieved by combusting additional fuel, like in catalytic flow-reversal reactors for methane from air ventilation from mines (VAM)^25^. On the contrary, photo-catalysts can convert the GHGs at temperatures close to room temperature^26,27^. Among other elements, TiO_2_ based photo-catalysts have been identified as suitable candidates for converting methane^28^. Successful trials have been conducted to reduce the odor and VOCs from exhaust of swine feedlots using TiO_2_ based photocatalysts^29^. One of the recent systems under development that uses the photo-catalyst is the solar chimney power plants (SCPP)^28^. In SCPP, the photo-catalyst is placed in the solar panels that generate electricity using solar energy while removing methane and nitrous oxide from air. A completely different method of CH_4_ mitigation involves spraying methanotrophic bacteria on sources such as trees or over wetlands to prevent the release of CH_4_ into the atmosphere. Other methods proposed in the literature include the generation of hydroxyl radicals and chlorine atoms for CH_4_ removal from point sources^30^.

The concept of CO_2_ capture from air (direct air capture) was first suggested by Lackner et al.^31^. Thereafter, the concept has received increased attention among researchers in various facets including materials and process development. Sorption based processes are among the most developed technologies for DAC. Particularly, amine-functionalized adsorbents are increasingly researched due to the low energy requirements for their regeneration and their potential for higher scalability^32^. Several reviews on the sorbents, process technologies, and socio-political challenges for implementation of DAC are published in the literature^33–36^.

The current paper presents a first-of-its-kind methane removal process using thermal- and photocatalytic route coupled to a CO_2_ capture unit. This work builds on the experimental studies on methane oxidation reported in the literature to design a process for methane conversion to CO_2_. The process for methane conversion is then coupled to a CO_2_ capture resulting in co-removal of methane and CO_2_ from low concentration streams. Since the concentration of methane in the streams from some of the agricultural sources and wetlands is higher than ambient air, the study has higher practical relevance to anthropogenic non-fossil methane emissions from agriculture sector and natural methane emissions from wetlands. We present the first-of-its-kind process concepts for co-removal of methane and CO_2_ and calculate the total energy demand per ton of CO_2_-equivalent (referred to as CO_2eq_ in the remaining text) removed. Much of the emphasis in this work has been on presenting the process concepts for CH_4_ removal and co-removal of CH_4_ and CO_2_ from the sources with elevated CH_4_ concentrations than present in the atmosphere. Therefore, the practical aspects with regards to installation of such devices have not been discussed in detail. Nonetheless, we envisage the following practical aspects regarding the installation of such devices. For installations on sources such as stable ventilation we consider that such a greenhouse gas removal device can be practically attached to the ventilation exhaust. For more open systems such as in wetlands or similar open agricultural sources, a challenge lies in capturing and converting the greenhouse gas before it is diluted in the atmosphere, and thereby the scale of the system will be similar (or bigger) to the current DAC technologies. This is partly addressed by placing the device close to emission sources. The technical challenges to overcome before implementation of the processes are discussed in the later sections, opening opportunities for future research.

## Methods

### Modelling assumptions—thermal catalytic route

A steady state thermodynamic model is modelled in Aspen Plus V12. To estimate the properties of the refrigerant (in dehumidifier) the physical property method inbuilt in Aspen Plus REFPROP is used, and NRTL-RK with default values is used as the global property method for the rest of the process. REFPROP is the suggested property method for refrigerants^37^. NRTL-RK closely estimates the dew point for the mixture of gases considered in the process to emulate air. Except for in the dehumidification unit where the condensation of water is expected, the rest of process is in vapor phase where Redlich-Kwong equation of state model is used as the property method from NRTL-RK. The inlet air is considered to be at 15 °C, 1 atm, and 60% relative humidity (RH)^38^. The detailed composition of inlet air is taken from Schubert et al.^39^ with the updated concentrations for CO_2_ and N_2_O from Global Monitoring Laboratory (GML) of the National Oceanic and Atmospheric Administration (NOAA)^40^. The updated inlet methane concentration is 300 ppmv representing the maximum concentration from the dairy ventilation air^5^. Key modelling assumptions are presented in Table 1.

**Table 1:** Key modelling assumptions in the process.

Component	Specification	Unit	Value	Reference
Blower	Polytropic efficiency	–	0.8	^41^
	Pressure increase	bar	0.25	–
Dehumidifier	Pressure drop (in each component)	% of inlet pressure	2	^41^
Compressor (dehumidifier)	Polytropic efficiency	–	0.8	^41^
	Compression ratio	–	7	–
Recuperator	$\Delta$ T_min_	°C	20	^41^
	Pressure drop (hot/cold side)	% of inlet pressure	2	^41^
Reactor	Pressure drop	% of inlet pressure	5	–
Heater	Pressure drop	% of inlet pressure	2	^41^
Pump (cooling water recirculation)	Efficiency	–	0.8	^41^
Cooler	Pressure drop (liquid side)	bar	0.4	^41^
	Pressure drop (gas side)	% of inlet pressure	2	^41^
	Minimum temperature difference ($\Delta$ T_min_)	°C	3	-
	Temperature rise in cooling	°C	12	^41^
Direct air capture unit	Pressure drop	% of inlet pressure	5	–

### Assumptions to model dehumidification unit in thermal catalytic route

Refrigerant type dehumidifier is modelled in the process^42^. In a refrigerant type of dehumidifier, the inlet moist air stream is cooled to below its dew point on the evaporator. The condensed moisture gives up its latent heat of condensation to the refrigerant in the evaporator. The evaporator is designed at − 10 °C, and ammonia is considered as the refrigerant. Pressure drop of the refrigerant in the evaporator is neglected and the refrigerant is assumed to leave the evaporator in a saturated vapor state at − 10 °C. The refrigerant is compressed in the compressor and is later cooled down to a saturated liquid state at the end of the condenser. Pressure drop of the refrigerant in the condenser is neglected. The cooled and dehumidified stream leaving the evaporator is heated up while cooling the refrigerant in the condenser. This way, the latent heat lost by the air stream in the evaporator is recovered as sensible heat in the condenser. Refrigerant is later expanded adiabatically to the inlet state in an expansion valve. The flow rate of refrigerant is set the way to lower the inlet stream temperature to 1 °C for H_2_O condensation in the dehumidifier. The temperature is chosen to prevent the freezing of condensed water. The flow rate of refrigerant is 0.00084 kg/hr. The percent of H_2_O condensed in the dehumidifier is ~ 48%.

### Assumptions to model thermal-catalytic reactor

A fixed bed reactor, assumed to be loaded with 6.5 wt% Pd/Al_2_O_3_, operating at steady state, is considered for methane oxidation. The reactor is simulated in isothermal conditions in accordance with the experimental data obtained for the catalyst. Steady state kinetics from the equations provided by Alyani et al.^43^, was modelled to obtain the conversion with the amount of catalyst used in the reactor. Currently, in the process modelled, 100% conversion of the methane is assumed. Although, it is theoretically possible to achieve such conversion as shown in the temperature-programmed oxidation (TPO) results obtained by Alyani et al.^43^, under steady state conditions the conversion is reduced due to catalyst deactivation. Because in this work we aimed to present a feasibility study for the co-removal of methane and carbon dioxide, the kinetic aspects of the methane oxidation reaction are not addressed. However, an analysis showing sensitivity to the conversion is conducted and is described in the results section. The tested methane concentrations in Alyani et al.^43^ are (i) 1000 ppmv in 20 vol% O_2_, and the balance Ar and He and (ii) 5000 ppmv in 20 vol% O_2_, 5 vol% H_2_O (and 0 vol% H_2_O), and the balance He. Although the inlet CH_4_ concentration considered in this work is 300 ppmv, we assume a similar catalytic performance as presented in Alyani et al.^43^ and complete conversion. A comprehensive techno-economic assessment for the process should give a more holistic view of the differences in opting between the amount of catalyst and the conversion rate.

Pressure drop in the isothermal reactor is assumed to be 5%. A sensitivity for the pressure drop is performed to highlight its impact on the total energy required for the process.

### Assumptions for CO_2_ capture unit

A CO_2_ capture unit to capture and remove the CO_2_ from the reactor outlet stream is coupled to the methane oxidation process. Vacuum temperature swing adsorption (VTSA) on solid sorbents is considered for the CO_2_ capture unit. Sabatino et al.^44^ presented direct air capture processes based on various liquid and solid sorbent systems. From among the models presented by Sabatino et al.^44^, VTSA based on APDES-NFC as the sorbent is considered in this process. The CO_2_ capture and compression unit was not separately modelled rather the energy obtained from already modelled system by Sabatino et al.^44^ was used in this paper. However, since the CO_2_ capture unit is coupled to the methane conversion process, an additional blower is not required. Therefore, the energy demand corresponding to the blower from the total energy estimated by Sabatino et al.^44^ is subtracted. The resulting energy demand for the CO_2_ capture step is 11.04 GJ/t-CO_2eq_.

### Assumptions for CO_2_ capture unit with heat integration:

**Figure 2:**
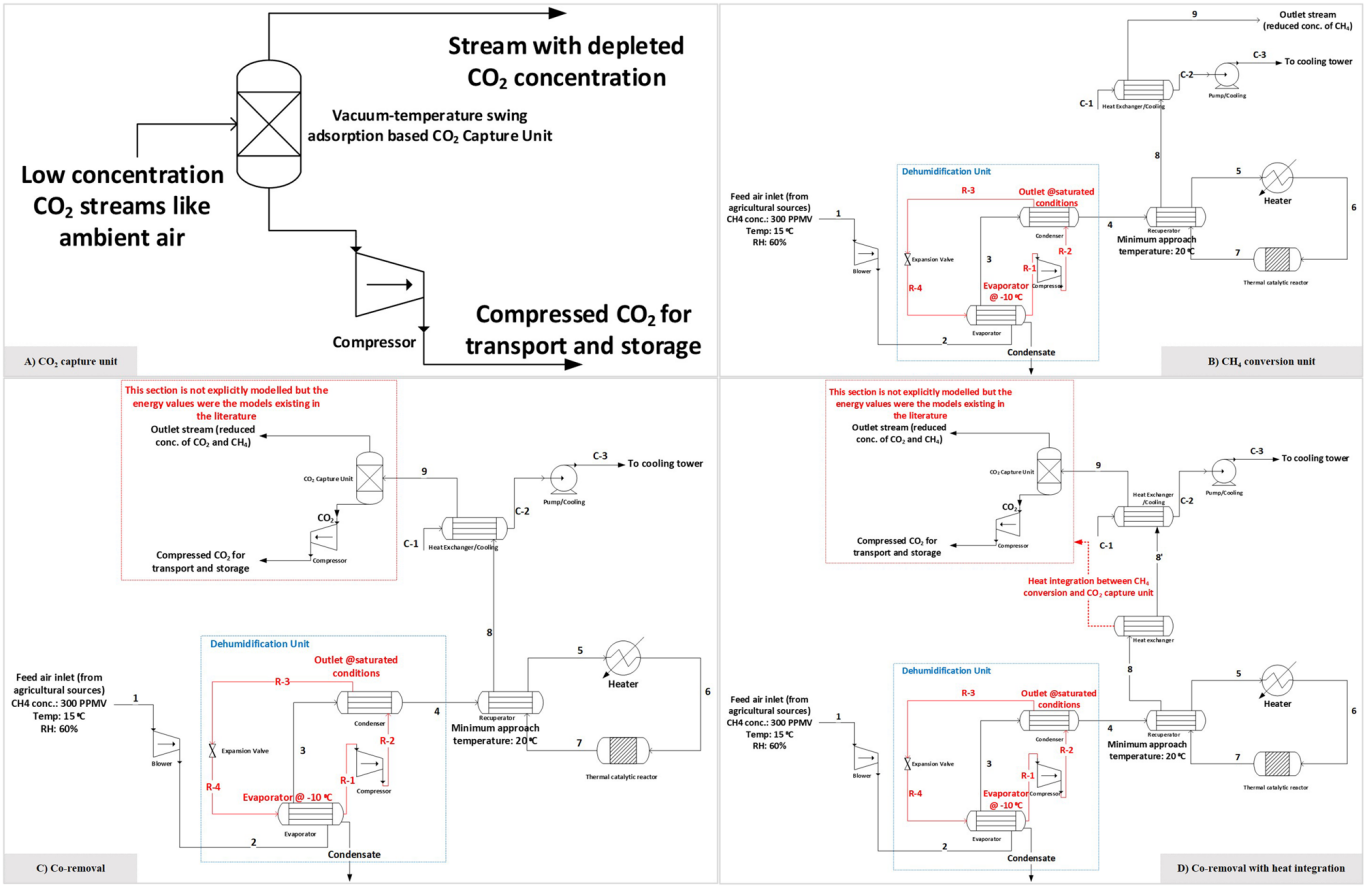
Process flow diagram of different cases by thermal catalytic route. (**A**) CO_2_ capture by vacuum temperature swing adsorption-based process. (**B**) Methane conversion. (**C**) Methane conversion with CO_2_ capture presented as co-removal. (**D**) Co-removal with heat integration between methane conversion and CO_2_ capture unit.

The process co-removal with heat integration considers that the thermal energy that is rejected in the cooler can be used to meet the thermal energy demand for adsorbent regeneration in the CO_2_ capture unit. This is assuming that the sorbent can be regenerated using the thermal energy of the CH_4_ depleted stream (stream 8 in the process flow chart in Fig. 2C @ 92 °C) from the recuperator, and that the regeneration can happen at about 80 °C (∆T: 10 K). The thermal energy of the stream between 92 and 80 °C will provide the heat for regeneration. For the CO_2_ capture process considered in this paper, the thermal energy demand in the desorption step is 10.1 GJ/t-CO_2_^44^. Since the desorption temperature was considered to be at about 80 °C instead of 100 °C in the referred CO_2_ capture process^44^, the vacuum pressure must be lower than considered (0.1 bar) to maintain the productivity. However, due to the lack of a detailed CO_2_ capture process model, this was not accounted for in the current work.

### Modelling assumptions—Photocatalytic route

The photocatalytic route was modelled in a similar way as the thermal route in Aspen Plus with most of the process units sharing the same assumptions. Pressure rise in the blower is adjusted according to the drop in rest of the process. Summary of the modelling methodology and assumptions of the other process units for the photocatalytic process are presented below.

### Assumptions to model cooler in photocatalytic route

A cooler is modelled that reduces the temperature of the stream from dehumidifier to 20 °C before being sent to the photocatalytic reactor. The minimum temperature difference of 3 °C is observed between hot stream outlet and the cooling water inlet.

### Assumptions to model photocatalytic reactor

Rstoic reactor in Aspen Plus was used to model the photocatalytic reaction. Isothermal conditions were assumed in the reactor. Pressure drop in the reactor was 5% of that of inlet pressure. The catalyst considered is 0.8 wt% CuO/ZnO. A conversion value of 92.5%, assumed in the reactor, is based on experiments on the photocatalyst presented in Li et al.^45^. The heat resulting from isothermal operation is assumed to be rejected, and no energy consumption is reported for the reactor. However, for the experiments on the photocatalyst in the said literature, a 300 W Xe lamp was used to simulate the sunlight. This resulted in an intensity of ~ 200 mW/cm^2^ in the reactor. Based on the data from Li et al.^45^, an estimation for the energy requirement for the photocatalytic reaction is made and included in the total energy consumption. While the energy source in a photocatalytic process is sunlight, the intensity and the spectral distribution are variable and can sometimes be insufficient to drive up the reaction. Considering this variation, the energy demand estimation is made with a fraction of artificial sunlight from being present 100% of the time to 0% of the time (100% sunlight). The energy required for simulated sunlight is estimated assuming the surface area required to complete the photocatalytic reaction. The reactor for artificial illumination followed the design of flow reactor considered by Li et al.^45^ (horizontal surface area: 6*2 cm^2^). The total energy demand for artificial illumination is given by 2.4 W (12 cm^2^ * 200 mW/cm^2^).

### Definition of key performance indicators:

The key performance indicator used for the presented process routes is the energy required to mitigate a ton CO_2_ equivalent of GHGs (t-CO_2eq_.). The CO_2_-equivalents corresponding to methane mitigation was obtained from GWP100 data from the IPCC fifth assessment report (AR5)^15^. The following relation presents the conversion of methane equivalents to CO_2_ equivalents:

1 $$\begin{array}{*{20}c} {X\; moles \;of\; CH_{4} = X \left( {{\text{mol}}} \right)*16 \frac{{g.{\text{CH}}_{4} eq.}}{{{\text{mol}}}}*28 \left( {\frac{{g.{\text{CO}}_{2} eq.}}{{g.{\text{CH}}_{4} eq.}} } \right) } \\ \end{array}$$

In the case without CO_2_ capture unit, the CO_2_ that is produced from the methane oxidation reaction is not captured. This reduces the overall equivalents of methane destruction. The following relation is then used to capture the reduction in CO_2_ equivalents for the case without the CO_2_ capture unit:

2 $$\begin{array}{*{20}c} {X \;mole \;of\; {\text{CH}}_{4} = X \left( {{\text{mol}}} \right)*\left( {16\left( {\frac{{g {\text{CH}}_{4} eq.}}{{{\text{mol}}}}} \right)*28\left( {\frac{{g {\text{CO}}_{2} eq.}}{{g {\text{CH}}_{4} eq.}}} \right) - 44 g {\text{CO}}_{2} eq.} \right)} \\ \end{array}$$

For the process model, the flow of 250 cc/min at the given inlet conditions were assumed. The energy demand is estimated from the simulations and was later extrapolated to a total of 1 t-CO_2eq_ removed from the system. The following relation is used in extrapolating to per ton of CO_2eq_ removed:

3 $$\begin{array}{*{20}c} {Total\; energy \;demand_{{t - CO_{2} eq}} \left( {\frac{GJ}{{tonne - {\text{CO}}_{2} eq}}} \right) = } \\ {\frac{{Total\; energy\; demand_{system} \left( W \right)}}{{Total \;{\text{CO}}_{2} eq. \;removed_{system} \left( {\frac{{{\text{kg}} - {\text{CO}}_{2} eq}}{s}} \right)*10^{6} }} } \\ \end{array}$$

The total energy demand is a combination of both the thermal and electrical energy demand in the process. The electrical heater is assumed to have 100% efficiency.

## Results

We discuss the energy demand in the two routes, thermal- and photo-catalytic, for methane conversion with and without CO_2_ capture. The total energy demand in the process is represented in GJ per t-CO_2eq_ removed within the system boundary depicted in Fig. 1.

### Thermal catalytic route

Methane oxidation to carbon dioxide through the thermal catalytic routes occurs typically at temperatures greater than 300 °C^20^ . Although we present thermodynamic analysis in this study, the choice of the catalyst is important for defining the process steps. A literature review was carried out to identify suitable thermal catalysts that can convert methane at concentrations less than 1%-vol. The identified catalyst formed the basis for proposing a process to convert methane and capture CO_2_ in the next step from the streams having low concentration of these gases (2 ppmv-1%-vol).

The main criteria to choose a catalyst is its ability to oxidize methane at concentrations less than 1%-vol in the streams, ease of synthesizing, and design temperatures at which > 90% methane is oxidized to CO_2_. Based on these criteria, we chose the results obtained from experiments performed by Alyani and Smith^43^ with 6.5 wt% Pd/Al_2_O_3_ as catalyst for oxidizing methane at low concentrations. The catalyst exhibits reversible inhibition to methane conversion on adding H_2_O to the feed gas^43^. This behavior is consistent with the inhibition displayed by PdO- based catalysts, which is partially attributed to slower desorption of H_2_O molecules from the catalyst surface^43,46–48^. As a result, the oxygen exchange between the support and the vacant sites is lowered, thereby reducing the regeneration capacity of vacant Pd-* sites. The elementary steps leading to the methane oxidation on adsorption over PdO are presented below^43,49,50^:

$${\text{CH}}_{4} + {\text{Pd}} - {\text{O}} + {\text{Pd}} -^{*} \to {\text{Pd}} - {\text{OH}} + {\text{Pd}} - {\text{CH}}_{3}$$

$$2{\text{Pd}} - {\text{OH}} \to {\text{H}}_{2} {\text{O}} + {\text{Pd}} - {\text{O}} + {\text{Pd}} -^{*}$$

$$2{\text{Pd}} -^{*} + {\text{O}}_{2} \to 2{\text{Pd}} - {\text{O}}$$

where Pd–* represent an O − vacancy

The standard heat of the reaction of complete methane oxidation reaction is − 891 kJmol^−1^, and the reaction is given by:

$${\text{CH}}_{4} + 2{\text{O}}_{2} \to {\text{CO}}_{2} + 2{\text{H}}_{2} {\text{O}}$$

The reduction of regeneration capacity was studied in the literature by measuring the effects of using supports with high oxygen storage capacity^43^, temperature, H_2_O concentration^51^, and the preparation method of catalyst^52^. The performance of Pd based catalysts is very sensitive to the H_2_O concentration in the feed streams. The concentration of H_2_O represents the humidity in the air stream we consider in our study. For a given inlet conditions and methane conversion rate, the amount of catalyst (g) at steady state varies linearly with RH^53^. We avoid the H_2_O induced inhibition by introducing a dehumidifier before the methane oxidation reactor to lower inlet H_2_O concentration. This is followed by a recuperator to pre-heat the inlet air. An additional heater is required to increase the air stream temperature to the design temperature in the reactor. We assume that the heater uses renewable electricity to pre-heat the stream to the design temperature in the reactor. Later, the feed is passed through the fixed bed reactor loaded with 6.5 wt% Pd/Al_2_O_3_, where methane conversion takes place under isothermal conditions. The resulting product gases go through a CO_2_ capture unit for CO_2_ separation after heat recovery. The CO_2_ capture process considered in our analysis is the solid sorbent based vacuum temperature swing adsorption process for DAC of CO_2_^44^ . The output from the CO_2_ capture unit comprises of pure CO_2_ stream, and an outlet stream of substantially reduced methane and CO_2_ concentration. A steady state thermodynamic model is developed in Aspen Plus V12. Key modelling assumptions and the process parameters are described in the methods section. The described process and its variants are shown in Fig. 2. These processes can be defined as follows: (i) Direct air capture of CO2 using the solid sorbent technology^44^ as shown in Fig. 2A (ii) Methane conversion to CO_2_ via thermal-catalytic route as shown in Fig. 2B (iii) Co-removal by methane conversion to CO_2_ integrated with CO_2_ capture (solid sorbent technology) as shown in Fig. 2C (iv) route with higher degree of heat integration to co-remove methane and CO_2_ as shown in Fig. 2D. The stream data of the process with co-removal case of CH_4_ and CO_2_ (Fig. 2C) is presented in supplementary material (refer Table S1 in supplementary material).

**Figure 3:**
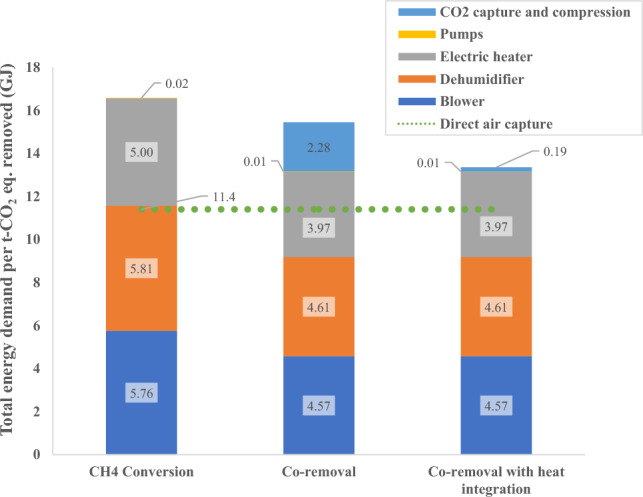
Energy consumption per t-CO_2eq_ removed for different cases.

Figure 3 compares the results for the total energy demand per t-CO_2eq_ removed for four different cases. The energy demand in the direct air capture (DAC) of CO_2_ is similar to one reported for solid sorbent based vacuum temperature swing adsorption technology and is equal to 11.4 GJ/t-CO_2_^44^. This is shown as horizontal green dotted line in the Fig. 3. The breakup of the energy demand from two main cases investigated in this work, only methane removal and co-removal, is shown by the bars on the left. The rightmost bar shows the energy demand with heat integration between methane conversion and CO_2_ capture unit. Assuming an air stream having 300 ppmv methane, the co-removal cases have ⁓7–20% lower energy demand than the methane conversion case for the same number of CO_2_-equivalents removed. The heat recovered from the cooling of the stream (stream without methane) leaving the recuperator and before entering the CO_2_ capture system, can be utilized to regenerate the sorbent in the CO_2_ capture unit. The estimated thermal energy of the stream from above 80 °C to be used for sorbent regeneration is 2.22 GJ/t-CO_2_ eq. This energy could potentially be used for sorbent regeneration in the CO_2_ capture process. In fact, the thermal energy requirement for CO_2_ capture per tonne CO_2_ equivalent removed in the co-removal/co-removal with heat integration case is 2.08 GJ. It is worth stressing here that this case highlights the potential for heat integration given the opportunity of heating the capture unit to about 80 °C for CO_2_ desorption or utilizing the rejected heat in pre-heating the water that is further converted to steam used in regeneration step. A detailed CO_2_ capture process model can closely simulate the intricacies of heat integration in CO_2_ desorption step and the potential tradeoffs in productivity it might have with desorption at different temperatures. Developing a detailed CO_2_ capture process model and its coupling with CH_4_ conversion unit will be a part of the future study, but the potential for such integration is discussed here. Another alternative is to utilize the thermal energy of the stream is to pre-heat the water which is later converted to steam for use in sorbent regeneration.

**Figure 4:**
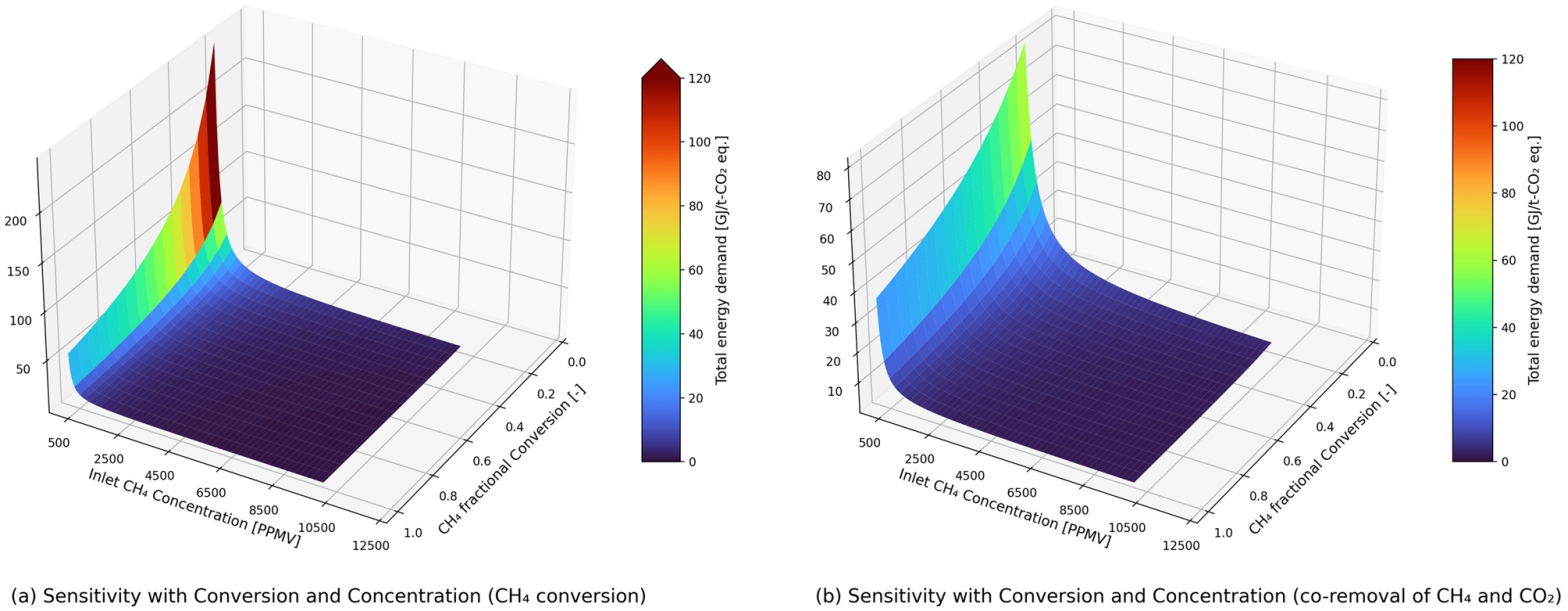
Figure showing sensitivity with both conversion and inlet methane concentration on the total energy demand for isothermal conditions in the methane conversion reactor.

Since we are discussing about the CO_2_-equivalents removed in the case of methane conversion/removal, both the methane concentration in the stream and its conversion in the reactor are very important to assess the process performance in terms of total energy demand. In Fig. 4, the sensitivity of the total energy demand with the inlet methane concentration and the methane conversion is presented for the cases with methane conversion and the co-removal of methane and CO_2_. The inlet methane concentration varies from 100 to 10,000 ppmv (in 50 ppmv steps) with the methane conversion varying from 0.2 to 1 (in 0.05 steps). The RH of inlet stream is maintained at 60% while performing sensitivity analysis with methane concentration.

At inlet methane concentrations of < 450 ppmv, the co-removal case has lower energy demand (per t-CO_2eq_ removed) than the methane conversion case. Figure 3 also confirms the same, where the assumed inlet concentration of methane was 300 ppmv. However, this trend changes for inlet methane concentrations > 450 ppm. At higher methane concentrations in the stream, just by converting the methane into CO_2_ removes more CO_2_-equavalents from the process per unit of energy, when compared to integrating it with a CO_2_ capture unit. This is because, in our analysis we have not captured the effect of CO_2_ concentration in the CO_2_ capture unit but have assumed a constant specific energy demand per ton of CO_2_ capture. In addition, the effect of RH on CO_2_ adsorption was not accounted for in the study. Oxidation of CH_4_ increases both the partial pressure of CO_2_ and H_2_O. The extent of this increase depends on the inlet CH_4_ concentration considered in the sensitivity study. For the base case with inlet CH_4_ concentration of 300 ppmv, the concentration of CO_2_ in the stream inlet to the capture unit and RH are 721 ppmv (partial pressure: 76.9 Pa) and 27%, respectively. The partial pressure of CO_2_ in the above sensitivity study varies from 47 to 1120 Pa, while the RH varies from 24 to 100% (H_2_O condensate is expected at higher CH_4_ concentrations). Due to the increase in CO_2_ adsorption capacity (mol_CO2_/kg_sorbent_) at higher CO_2_ partial pressures (as compared to 400 ppmv (~ 40 Pa) in the reference article^44^), an increase in CO_2_ adsorption is expected. Under the same desorption conditions, the working capacity will be higher, leading to a higher CO_2_ capture. The increase is, however, not reflected in the current work. It is reported that the water can increase CO_2_ adsorption in amine-based sorbents^44^. With increasing concentration and conversion, the partial pressure of CO_2_ and RH will both increase which might increase the productivity. However, the energy demand for the desorption step could also increase for the case with higher water co-adsorption. It is to be noted that the temperature is kept constant at 20 °C in line with the temperature considered in the reference CO_2_ capture process^44^. The validity of the fixed energy demand for CO_2_ capture in the sensitivity study is thus perhaps limited to CO_2_ concentrations close to 400 ppmv, i.e., between 100 and 300 ppmv (base case) of inlet CH_4_, and better at lower CH_4_ conversion rates. With competing interplay between CO_2_ partial pressure, RH, and energy demand, it is difficult to quantitively ascertain the extent of validity of the constant energy demand for CO_2_ capture considered in the study. For the case with minimum energy, Sabatino et al.^44^ reported, for the same sorbent, an energy demand of 9.7 GJ/t-CO_2_ (9.3 GJ/t-CO_2_—without blower). A sensitivity study on the co-removal energy demand considering the limiting cases and the base case revealed a decrease of approximately 2% for 100 ppmv and 300 ppmv, a decrease of 11% for 10,000 ppmv of inlet CH_4_ concentration. Approximately 16% decrease in energy demand from 11.04 GJ to 9.3 GJ has translated into only a 2% reduction in co-removal for the base case. As CO_2_ capture accounts for only about 20% of total CO_2_ equivalents removal in the base case, the effect due to its variability is less pronounced in sensitivity. The total energy demand is therefore less sensitive to the energy demand in the CO_2_ capture and compression unit. To give a perspective, the total energy demand in the methane conversion and co-removal case is 2487 and 115 GJ/t-CO_2eq_ removed if ambient air having 2 ppmv methane is treated in the process. For the air coming from stable ventilation (in agriculture farms), which is having methane concentrations between 10 and 300 ppmv, the total energy demand in the methane conversion and co-removal case is 17–497 and 15–97 GJ/t-CO_2eq_ removed (inversely related to concentration). For air from manure storage headspace having 10,000 ppmv methane sent to the process, the energy demand for the methane removal and the co-removal cases are ~ 0.5 GJ/t-CO_2eq_ and ~ 1.57 GJ/t-CO_2eq_ removed. For the air above wetlands, which can have methane concentrations between 7 and 40 ppmv^54^, the total energy demand in the methane conversion and co-removal case is 127–711 and 62–103 GJ//t-CO_2eq_ removed respectively (total energy demand is inversely related to concentration). However, the total energy demand in the solid sorbent-based DAC unit for CO_2_ capture is 11.4 GJ/t-CO_2_.

Figure 4 also shows that total energy demand in the process is sensitive to methane conversion. Therefore, the choice of catalysts to achieve higher conversion of methane in the reactor is important to have lower energy demand per t-CO_2eq_ removed from the process. The analysis in this work is based on 6.5 wt % Pd on alumina that has shown to achieve ⁓100% conversion (initial activity) of methane at 330 °C under dry conditions^43^. However, the presence of water vapor (RH) in the stream inhibits the methane conversion over the catalyst. Therefore, dehumidification step is included in the process flow, which contributes to 30–35% of the total energy demand in both the methane conversion and co-removal cases. Hence catalysts that are less sensitive to the presence of water vapor in the stream that can avoid the dehumidification step will help in reducing the total energy demand significantly.

**Figure 5:**
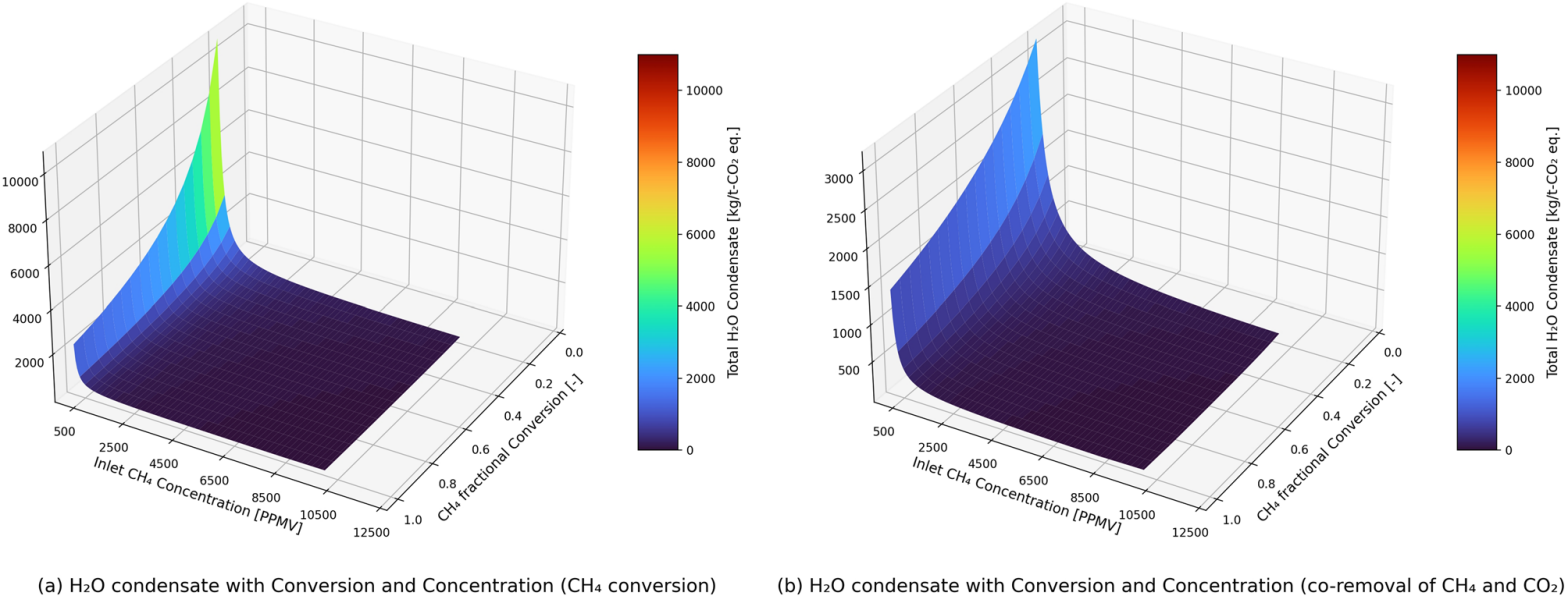
Sensitivity of inlet methane concentration and conversion on total H_2_O condensate from the process for the case with (**a**) methane conversion and (**b**) co-removal of methane and CO_2._ The methane conversion in the reactor is isothermal.

The amount of water condensate per t-CO_2eq_ is 715 kg for methane conversion case and 568 kg for co-removal in the reference case with 300 ppmv of methane concentration. The water condensate includes both the condensate from the dehumidifier and the expected condensate in the heat exchanger before CO_2_ capture unit. The latter, which is from methane oxidation, is only present at high inlet methane concentrations. Figure 5 presents the variation of water condensate with inlet methane concentration and conversion in the reactor. The water condensate per t-CO_2eq_ decreases with increasing inlet methane concentration and methane conversion in the reactor. This is due to increasing CO_2eq_ removal with the methane concentration and conversion while the water condensate is relatively constant.

**Figure 6:**
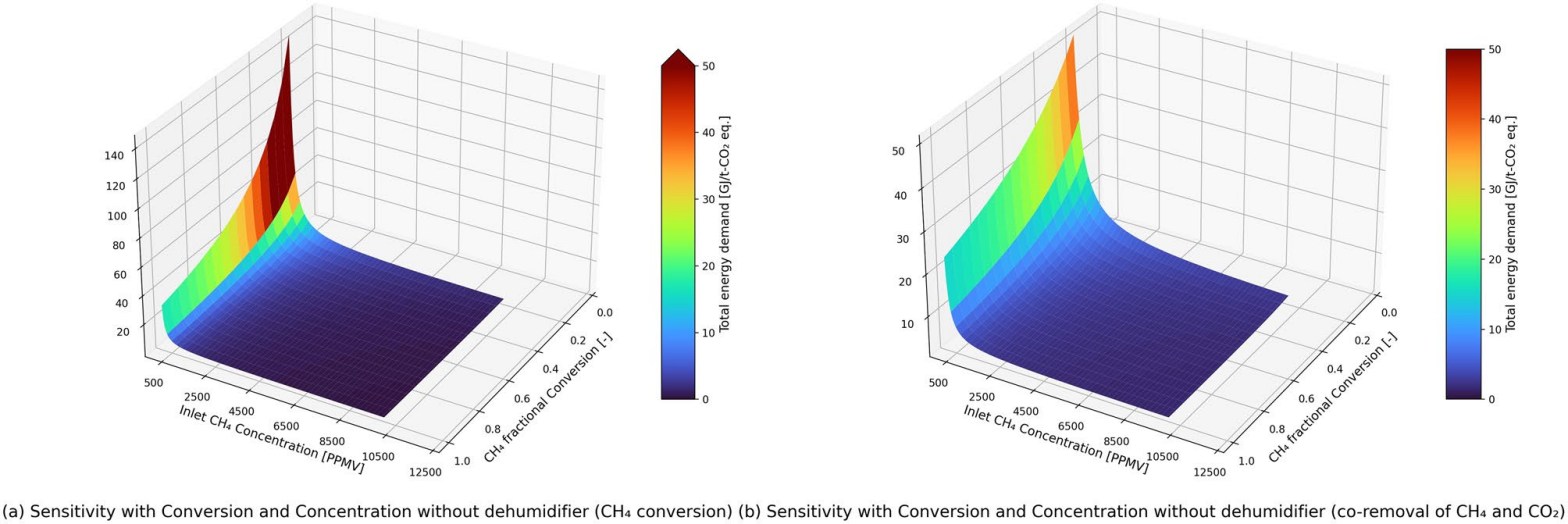
Sensitivity of total energy demand in the process with inlet methane concentration and methane conversion over a hypothetical catalyst that is not sensitive to water vapor concentration for the (**a**) methane conversion process without dehumidifier (**b**) co-removal process without dehumidifier.

In Fig. 6, we present the case for a hypothetical catalyst that is less sensitive to the presence of water vapor in the stream, thereby avoiding the dehumidification step in the process. Assuming 100% conversion (isothermal conditions) for a stream containing 300 ppmv methane, the total energy demand in the methane conversion and co-removal cases is 9.7 and 10 GJ/t-CO_2eq_ removed. Similarly, for 1%-vol of methane in the inlet (and 100% conversion), the process without dehumidifier has an energy demand of ~ 0.3 GJ/t-CO_2eq_ for methane conversion case, and ~ 1.4 GJ/t-CO_2eq_ for co-removal case. Therefore, in this case, the total energy demand to remove CO_2_-equivalents is significantly lower than the energy penalty in the state-of-the-art direct air capture of CO_2_^44^. Studies showed improved water resistance to catalytic methane oxidation from different oxide supports and their effects^43,55,56^. Through a different approach, Huang et al.^57^ reported a significant increase in the activity of Pd-based catalysts by using in situ water sorbents. The sorbent must be regenerated in the latter before using it in another cycle.

**Figure 7:**
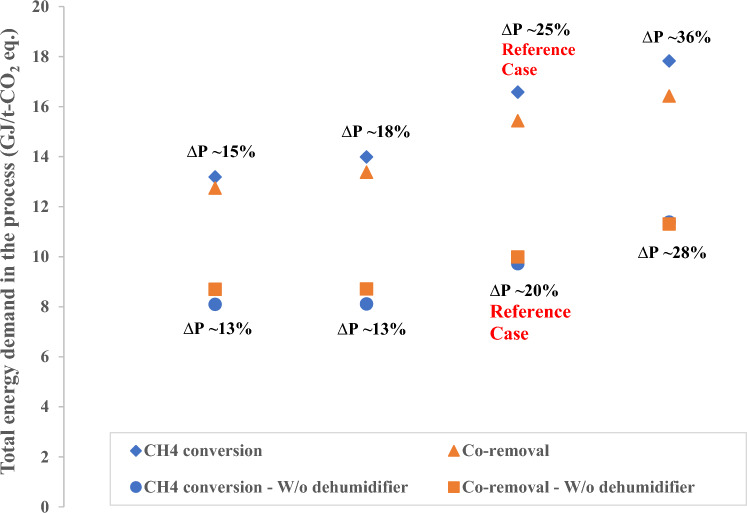
Graph presenting the sensitivity with total pressure drop in the system. The legend “W/o dehumidifier” denotes the process without dehumidifier.

The sensitivity with total pressure drop is shown in Fig. 7. The following cases were studied in the sensitivity:

1. Reference case refers to the case with 2% pressure drop (of the respective component inlet pressure) for all the components except reactor and CO_2_ Capture unit; for the reactor and CO_2_ Capture unit the pressure drop is 5% of their inlet pressure. The total pressure drop in the entire process is ~ 25% of the atmospheric pressure.
2. The other three cases represent total pressure drop in the system of ~ 15%, ~ 18%, and ~ 36% respectively. The background for these cases is that for case with total pressure drop of 15%, the pressure drop in all the components is 1% of their respective inlet pressure except for the reactor and CO_2_ Capture unit where the pressure drop is 4% of their inlet pressure. For the case with total pressure drop of 36%, the pressure drop is 3% of inlet pressure for all the components except the reactor and CO_2_ Capture unit where the pressure drop is assumed to be 6% of their inlet pressure. Case with 18% pressure drop represents the systems where the pressure drop in all the components is 2% of their respective inlet pressure.

In Fig. 3, we observe that the blower contributes to 30–35% of the total energy demand in methane conversion and co-removal cases. The blower is responsible for increasing the pressure of inlet air stream by considering the pressure drop in each unit step of the process. Therefore, the total energy demand is proportional to the overall pressure drop in the process, as seen in Fig. 7. The pressure drop values considered in our analysis are very conservative, and the actual pressure drops will be lower than assumed in the analysis. For example, we assume a 2–6% pressure drop (21–65 mbar) across the solid sorbent-based CO_2_ capture unit, but it has been reported that the pressure drop is only 1 mbar^58^. The case without a dehumidifier will have a lower overall pressure drop in the process, therefore the energy demand is lower. We observe that an increase in 1% pressure drop from across all the process steps in both the methane conversion and co-removal cases will lead to increase of total energy demand by > 20% when the initial pressure drop is 1% for all the units and 4% for the reactor and CO_2_ capture unit.

**Figure 8:**
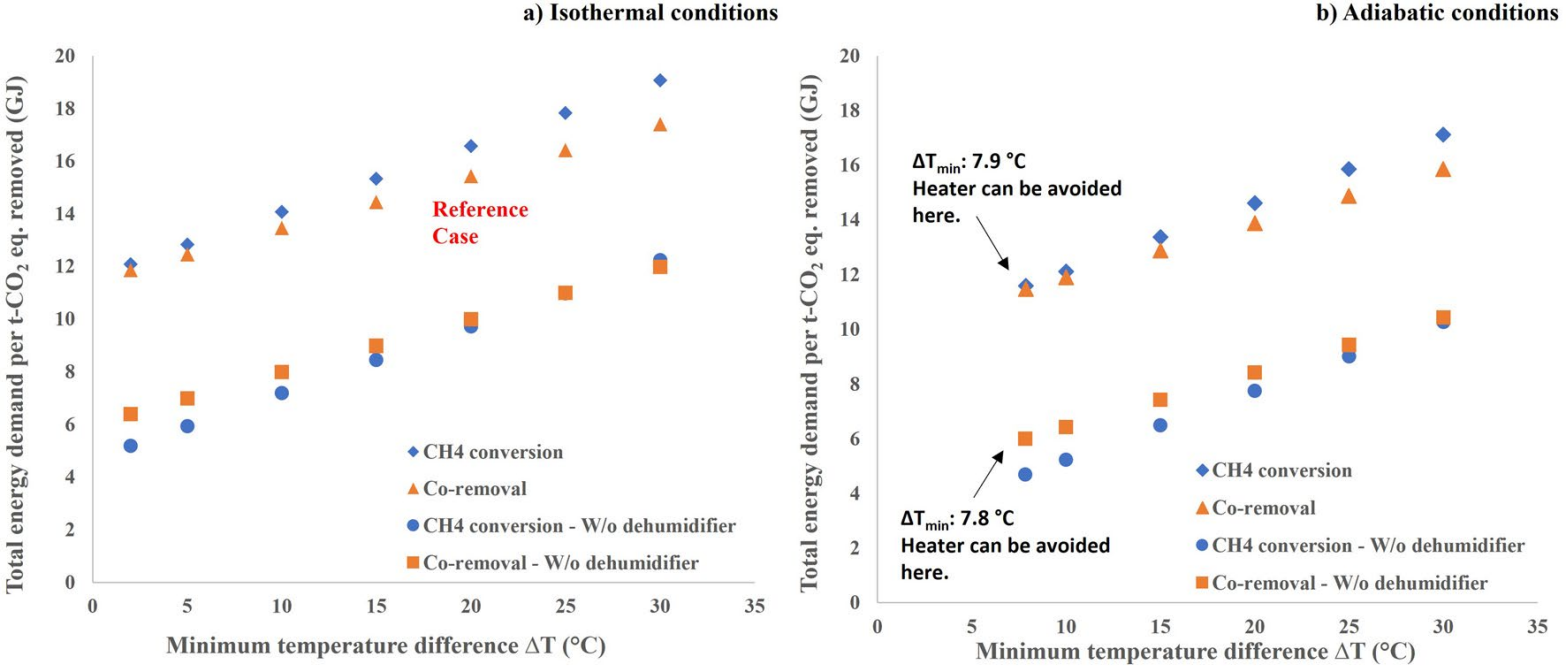
Plots showing the sensitivity on total energy demand with respect to minimum temperature difference in the recuperator. Sensitivity plot with reactor at isothermal conditions (**a**) and at adiabatic conditions (**b**). The legend “W/o dehumidifier” denotes the process without dehumidifier.

The heater in the process contributes to 25–30% of the total energy demand in the process (as seen in Fig. 3). This heater pre-heats the reactor feed to the desired reaction temperature in the reactor. The energy demand in the heater can be optimised in different ways that are (i) using the catalyst that can achieve higher conversion at lower temperatures (ii) having a lower minimum approach temperature (∆T_min_) in the recuperator (iii) designing the reactor for adiabatic conditions to utilise the heat of the methane oxidation reaction. Figure 8a and b shows the total energy demand for isothermal and adiabatic design conditions in the reactor for different ∆T_min_ in the recuperator. Designing a recuperator with lower ∆T_min_ will result in lower total energy demand in the process. However, there will be a trade-off between ∆T_min_ and heat exchanger’s size and costs.

Considering 300 ppmv methane concentration in the inlet stream and the reactor designed for isothermal conditions, the total energy demand can be 23–27% lower when the ∆T_min_ is 2 °C when compared to the scenario with ∆T_min_ of 20 °C in the recuperator. The scenario with a hypothetical catalyst that is not sensitive to the presence of water vapor, the total energy demand in the process is 5–6 GJ/t-CO_2eq_ removed when the ∆T_min_ is 2 °C in the recuperator. When designing the reactor for adiabatic conditions, the heat from methane oxidation increases the reactor temperature, thereby having the product stream leaving at a higher temperature than the inlet. In such scenarios, with inlet methane concentration of 300 ppmv, a ∆T_min_ of 7.8 °C in the recuperator is enough to eliminate the need of the heater. In this case, the total energy demand is between 5 and 12 GJ/t-CO_2eq_ and 6–11 GJ/t-CO_2eq_ for the methane conversion and co-removal cases respectively, depending on if we need a dehumidifier or not. The total energy demand in the adiabatic case is lower than the isothermal case. Adiabatic case is also advantageous since the heat from methane oxidation remains within the system leaving more heat that can be potentially utilized to regenerate the sorbent in the CO_2_ capture unit.

Considering all the favorable design conditions in the process, which is having lower pressure drops (1% at each step of the process), adiabatic conditions for the methane conversion reactor, ∆T_min_ as 2 °C or eliminating the need of the heater, the catalyst being robust to the water vapor in the stream thereby avoiding the need of the dehumidifier, the total energy demand for methane conversion and co-removal is:

- 302 and 23 GJ/t-CO_2eq_ for ambient air having 2 ppmv methane
- 1.5 and 3.5 GJ/t-CO_2eq_ for air from stable ventilation (with 300 ppmv methane)
- 86–13 and 21–12 GJ/t-CO_2eq_ for air above the wetlands (with 7–40 ppmv methane)
- 0.05 and 1.16 GJ/t-CO_2eq_ for air above manure storage pits (with 10,000 ppmv methane)

### Photocatalytic route to convert methane

**Figure 9:**
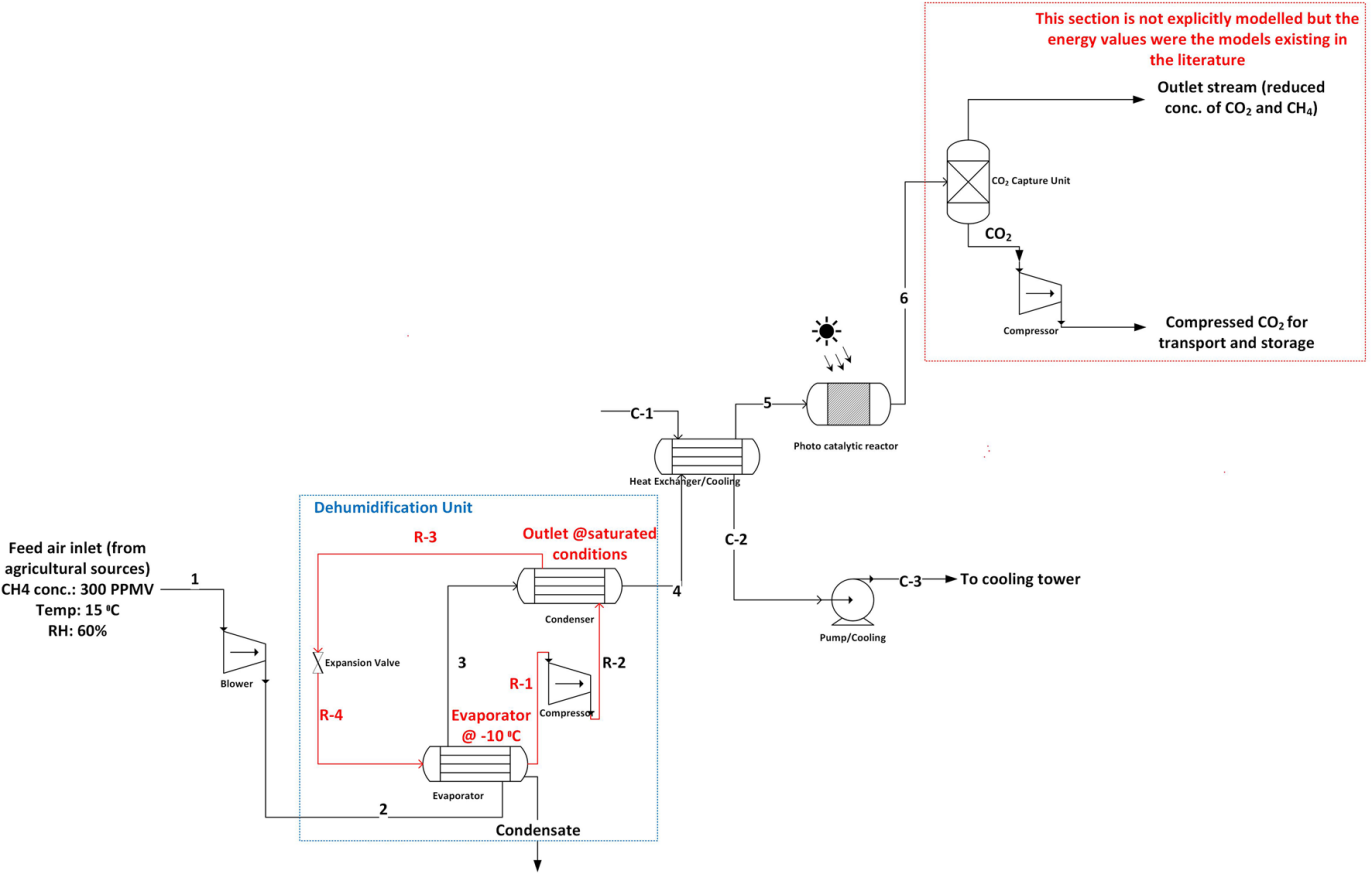
Process flowsheet of CH_4_ conversion with CO_2_ capture by photocatalytic route.

The process flowsheet for the photocatalytic route is shown in Fig. 9. Similar to the thermal catalytic route, stream with low concentration of methane passes through a blower and later goes through a dehumidification step. The dehumidified stream is cooled to the ambient temperature (20 °C) in a cooler. This is followed by the photocatalytic conversion of methane in a reactor. The stream is now richer in CO_2_ from the methane conversion. From literature, catalyst with good photocatalytic activity for low concentration methane conversion was assumed, to define the methane conversion (92.5%). Li et al.^45^ presented 0.8 wt% CuO/ZnO nanocomposite photocatalyst for methane oxidation as one of the cost effective alternatives to photocatalysts based on noble metals. A CO_2_ capture unit later separates the CO_2_ from the stream and is sent for compression, transport, and storage. The energy estimation for CO_2_ capture follows the same methodology presented in the thermal route. The modelling of CO_2_ transport, and storage is out of the scope of this work.

**Figure 10:**
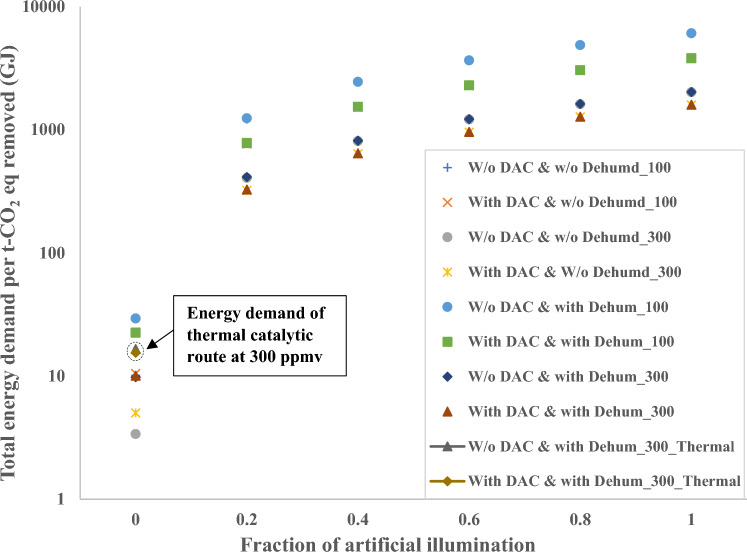
Graph showing the total energy demand for photocatalytic route for inlet methane concentrations of 100 ppmv and 300 ppmv. W/o DAC indicates without CO_2_ capture unit, and w/o_dehum indicates without dehumidifier.

Figure 10 presents the total energy demand in methane conversion process and the co-removal process via photocatalytic route. The analysis is carried out for inlet methane concentrations of 100 ppmv and 300 ppmv, since the modelling study is based on experiments conducted by Li et al.^45^ over a 0.8 wt% CuO/ZnO photocatalyst for 100 ppmv methane in 78.9% N_2_ and 21.1% O_2_. We use similar conversion efficiency for the case with 300 ppmv, mainly to compare the results with thermal catalytic route. Figure 10 also presents the sensitivity of process performance with respect to the artificial illumination required in the process. The fraction of artificial solar light illumination was varied from 0 to 1 in steps of 0.1 to present the spectrum of energy demand due to the natural variation of sunlight. It is assumed that the electrical energy demand for providing illumination to simulate artificial sunlight is converted entirely into the intensity (~ 200 mW/cm^2^) that reaches the reactor. As seen in Fig. 10, total energy demand is highly sensitive to the fraction of artificial solar illumination. For the case with an inlet methane concentration of 300 ppmv and without any artificial solar illumination, the energy demand is 9.8 GJ/t-CO_2eq_ and 10 GJ/t-CO_2eq_ for methane conversion and the co-removal processes respectively. This is nearly 35% lower than the thermal catalytic route. However, with 100% artificial illumination the energy demand for the same case is approximately 2000 GJ/t-CO_2eq_ and 1600 GJ/t-CO_2eq_ for methane conversion and the co-removal processes respectively. The photocatalytic route, considering only methane conversion (300 ppmv in the inlet stream) can reach a total energy demand of ⁓3 GJ/t-CO_2eq_ if the process does not require a dehumidifier and artificial solar illumination. For the process to be effective, and in a more optimistic scenario, locations with a higher solar irradiance should be preferred for the photocatalytic route, or the process should be run when the sunlight is available (during the day).

## Discussion

In this section, we briefly discuss the total energy demand in thermal- and photocatalytic routes in comparison with the reference CO_2_ capture process. The DAC process modelled in Sabatino et al.^44^ with a sorbent similar to the one used by Climeworks is considered as the reference CO_2_ capture process. The energy demand for CH_4_ conversion and co-removal cases in the thermal catalytic route are 1.5 and 1.4 times the energy demand of the reference CO_2_ capture. With 80% CO_2_ equivalent removal from just CH_4_ conversion in the co-removal case, CH_4_ conversion contributes significantly to the total energy demand. This is reflected in lower energy demand for co-removal case when compared to the CH_4_ conversion route. For the case without dehumidifier, CH_4_ conversion has a lower energy demand than the reference CO_2_ capture energy demand. This results in higher energy demand for the co-removal case. The energy demand per t-CO_2eq_ removed in the thermal catalytic route without dehumidifier for CH_4_ conversion and co-removal are 0.85 and 0.88 times the total energy demand in the reference CO_2_ capture, respectively.

The CH_4_ conversion process in thermal catalytic route is similar to the technologies employed to destroy ventilation air methane in coal mines. However, those technologies have a minimum concentration limit (> 1000 ppmv), and higher design temperatures (for thermal flow reversal reactors) to allow for the continuous autothermal operation of flow reversal reactors^59^. Therefore, these technologies as such do not need a major energy supplement while under established continuous operation above a threshold and stable CH_4_ concentrations. The difference in our process can be seen in that at lower concentrations (such as 300 ppmv), an additional energy demand is expected to sustain the conversion process in a way similar to sustain an autothermal reaction at concentrations lower than the threshold concentration for autothermal reaction. For instance, a catalytic flow reversal reactor for ventilation air methane in coal mines, a threshold CH_4_ concentration of 0.1 vol% is required to provide sufficient heat to sustain the reaction^59,60^.A catalytic monolith reactor (CMR) with a recuperator has a similar threshold CH_4_ concentration of 0.4%^59^. However, in this study, given a well-established H_2_O inhibition characteristic to CH_4_ catalytic oxidation with noble metal catalysts^61^, a dehumidifier or a similar H_2_O removal method is required. This adds to an additional energy demand in the thermal catalytic route.

Photocatalytic route (without artificial illumination) at 300 ppmv of inlet CH_4_ concentration has an energy demand of 0.86–0.88 times and the case without dehumidifier is 0.3–0.4 times the energy demand of the reference CO_2_ capture (solid sorbent based direct air capture technology). The energy demand of photocatalytic route, however, increases drastically if we require artificial illumination.

The current state-of-the-art CO_2_ capture process has an energy requirement of ~ 1.3 times the reference CO_2_ capture process considered in this study^44^. On the other hand, the minimum work of separation of CH_4_ from atmosphere (1.88 ppmv) with 70% capture rate and 97% purity is 62 MJ/t-CO_2_eq (GWP100: 34)^62^. With a GWP100 of 28 (the value considered in this work) and with the same capture rate and purity as the former, the minimum work of CH_4_ separation from a point source with 300 ppmv is 47 MJ/t-CO_2_eq. The exergy demand for the thermal route is estimated to be 17 GJ/t-CO_2_ eq. It is to be noted that the minimum work of CH_4_ separation and the exergy required for CH_4_ conversion are not to be compared. However, we see scope for exergy reduction in the heater which accounts for about 30% of total exergy demand, and dehumidifier, which is based on refrigerant has a share of about 35% of total exergy demand.

## Conclusions and outlook

In this article, we propose a first-of-its kind solution to address the challenge of removing methane from the atmosphere and low concentration non-fossil sources (methane concentration < 1%-vol). The solution is a combination of methane oxidation to CO_2_ over a catalytic filter and CO_2_ capture unit (similar to direct air capture). Methane oxidation to CO_2_ can be done using thermal- or photo-catalytic routes. CO_2_ can be captured using state-of-the-art solid sorbent technologies suited for low concentration sources. The proposed solution to co-remove methane and CO_2_ from low concentration sources has the potential of removing more CO_2_-equivalents from the atmosphere at lower energy penalty when compared to technologies that focus on removing only CO_2_ from air. Based on our analysis in this article, we summarize the following challenges and opportunities with the proposed solution for co-removing methane and CO_2_ from the atmosphere.

- Methane conversion and co-removal of methane and CO_2_ is a possibility from low concentration sources, but the total energy demand is very sensitive to the concentration of methane in the source. At lower concentration of methane (< 450 ppmv methane), co-removal is more energy efficient than just converting methane into CO_2_. However, for higher concentration of methane (> 450 ppmv), just converting methane into CO_2_ could be enough to efficiently remove CO_2_-equivalents. Placing the co-removal process closer to the source of methane emissions provides an opportunity to convert methane and co-remove methane and CO_2_ at a lower energy penalty than the process that only removes CO_2_ from ambient air. These places can be for example: air from stable ventilation, air above the manure storage headspace or even air above the wetlands where the methane concentration is more than in ambient air.
- Several challenges need to be overcome for the co-removal process to be energy efficient to remove CO_2_-equivalents from the atmosphere. These are with respect to materials (catalysts) that can convert methane at low concentration in air, heat exchanger design, reactor conditions and design, and heat integration with the CO_2_ capture step. Another challenge is treating other impurities like ammonia and volatile organic compounds (VOCs) and dust that may inhibit the catalyst. These studies need further investigation.
- Photocatalytic route has the potential of removing methane at lower energy penalty if artificial illumination can be eliminated, and the catalyst is effective in the presence of direct sunlight. However, the process is limited to the place and time for the sunlight.
- Although this article does not provide a life cycle analysis (LCA) for the proposed concepts, LCA will decide if the process will enable achieving negative emissions or not. Similarly, a techno-economic analysis will help in understanding the viability of the proposed concepts.
- Co-removal process also presents potential for process intensification where the methane conversion and CO_2_ capture can be done in a single step, or methane and CO_2_ can be converted to valuable products in a single step. These processes can also be integrated with nitrous oxide removal/conversion, where nitrous oxide can be converted to nitrogen and oxygen.
- The proposed concepts have the potential to remove 40% of anthropogenic non-fossil and 40% of natural methane from the atmosphere. The agricultural sector is likely to be the first mover of technologies since the concentration of methane in sources like stable ventilation and manure storage is significantly higher than the methane concentration in the ambient air.
- The scope of this work is limited to non-fossil methane, and we have avoided any methane coming from fossil sources, for example, leaks in the natural gas value chain or ventilation air in coal mines.

### Supplementary Information

Supplementary Information.

## Data Availability

The stream data generated for the co-removal process (Fig. 2C) is available in supplementary material.
