# Supplementary material for: Process concepts and analysis for co-removing methane and carbon dioxide from the atmosphere
Source: Sci Rep. 2023 Oct 12;13:17290. doi: 10.1038/s41598-023-44582-w (PMC10570372; doi:10.1038/s41598-023-44582-w)
Supplement: Supplementary file 1 — Supplementary Information. [file 41598_2023_44582_MOESM1_ESM.pdf]

Process concepts and analysis for co-removing methane and carbon dioxide from the atmosphere

Devesh Sathya Sri Sairam Sirigina[a*], Aditya Goel[b,c], Shareq Mohd Nazir[a**]

[a]Department of Chemical Engineering, KTH Royal Institute of Technology, Stockholm — 11428, Sweden

[b]Department of Chemical Engineering, Columbia University, New York, New York – 10027, USA

[c]Department of Chemical Engineering, Birla Institute of Technology and Science, Pilani — Goa Campus, Goa – 403726, India

Corresponding authors – *Devesh Sathya Sri Sairam Sirigina, sirigina@kth.se

**Shareq Mohd Nazir, smnazir@kth.se

Table S 1 contains the stream data for the co-removal case of the process flow show in Figure 2.

Table S 1: Stream data of the process with CO_2 capture unit (co-removal).

Stream no.	Volumetric flow rate (l/min)	Temperature (°C)	Pressure (bar)	CO_2 mol%	CH_4 mol%	H_2O mol%	Rem. mol%
**1**	0.25	15	1.01	0.04	0.03	1.01	98.91
**2**	0.22	38.97	1.27	0.04	0.03	1.01	98.91
**3**	0.19	1.00	1.24	0.04	0.03	0.53	99.39
**4**	0.25	70.81	1.22	0.04	0.03	0.53	99.39
**5**	0.43	310.00	1.19	0.04	0.03	0.53	99.39
**6**	0.45	330.00	1.17	0.04	0.03	0.53	99.39
**7**	0.48	330.00	1.11	0.07	0.00	0.59	99.33
**8**	0.29	91.62	1.09	0.07	0.00	0.59	99.33
**9**	0.24	20.00	1.07	0.07	0.00	0.59	99.33
**R-1**	0.01	-10.00	2.91	-	-	-	-
**R-2**	<0.01	228.62	20.34	-	-	-	-
**R-3**	<0.01	50.03	20.34	-	-	-	-
**R-4**	<0.01	-10.00	2.91	-	-	-	-
**C-1**	<0.01	17.00	2.92	-	-	100.00	0.00
**C-2**	<0.01	29.00	2.52	-	-	100.00	0.00
**C-3**	<0.01	29.00	2.92	-	-	100.00	0.00
**COND**	<0.01	1.00	1.24	0.00	0.00	100.00	0.00
